# Nitrogen-Doped Graphene: The Influence of Doping Level on the Charge-Transfer Resistance and Apparent Heterogeneous Electron Transfer Rate

**DOI:** 10.3390/s20071815

**Published:** 2020-03-25

**Authors:** Maria Coros, Codruta Varodi, Florina Pogacean, Emese Gal, Stela M. Pruneanu

**Affiliations:** 1 National Institute for Research and Development of Isotopic and Molecular Technologies, Donat Street, No. 67-103, 400293 Cluj-Napoca, Romania; codruta.varodi@itim-cj.ro (C.V.); florina.pogacean@itim-cj.ro (F.P.); 2 Department of Chemistry and Chemical Engineering, Hungarian Line of Study, Babes-Bolyai University, 11 Arany János St., 400028 Cluj-Napoca, Romania; gal.emese.81@gmail.com

**Keywords:** nitrogen-doped graphene, charge-transfer resistance, apparent heterogeneous electron transfer rate, modified electrodes, detection of 8-OHdG

## Abstract

Three nitrogen-doped graphene samples were synthesized by the hydrothermal method using urea as doping/reducing agent for graphene oxide (GO), previously dispersed in water. The mixture was poured into an autoclave and placed in the oven at 160 °C for 3, 8 and 12 h. The samples were correspondingly denoted NGr-1, NGr-2 and NGr-3. The effect of the reaction time on the morphology, structure and electrochemical properties of the resulting materials was thoroughly investigated using scanning electron microscopy (SEM) Raman spectroscopy, X-ray powder diffraction (XRD), Fourier-transform infrared spectroscopy (FTIR), X-ray photoelectron spectroscopy (XPS), elemental analysis, Cyclic Voltammetry (CV) and electrochemical impedance spectroscopy (EIS). For NGr-1 and NGr-2, the nitrogen concentration obtained from elemental analysis was around 6.36 wt%. In the case of NGr-3, a slightly higher concentration of 6.85 wt% was obtained. The electrochemical studies performed with NGr modified electrodes proved that the charge-transfer resistance (R_ct_) and the apparent heterogeneous electron transfer rate constant (K_app_) depend not only on the nitrogen doping level but also on the type of nitrogen atoms found at the surface (pyrrolic-N, pyridinic-N or graphitic-N). In our case, the NGr-1 sample which has the lowest doping level and the highest concentration of pyrrolic-N among all nitrogen-doped samples exhibits the best electrochemical parameters: a very small R_ct_ (38.3 Ω), a large K_app_ (13.9 × 10^−2^ cm/s) and the best electrochemical response towards 8-hydroxy-2′-deoxyguanosine detection (8-OHdG).

## 1. Introduction

Graphene, a single layer of graphite, possesses various interesting properties such as large surface area, high thermal and electrical conductivity and good optical transparency [1,2,3]. Currently, the chemical doping of graphene is an active area of research which continues to grow very quickly. Doping graphene with heteroatoms such as nitrogen [4,5], boron [6,7], sulfur [8,9] or halogens [10,11] can adjust the electronic and electrochemical properties of the material leading to enhanced performances. Among the heteroatoms, nitrogen has drawn a lot of attention due to the significantly improved properties of the N-graphene as part of fuel cells [12], lithium ion batteries [13], supercapacitors [14] or advanced catalyst support [15,16]. Nitrogen doping can be achieved using different approaches: mixing graphene with nitrogen containing precursor and thermal annealing [17,18]; thermal exfoliation of graphite oxide in ammonia atmosphere [19]; solvothermal or hydrothermal methods using graphene oxide and Shift base precursors [20,21,22], chemical vapor deposition [23] or microwave plasma [24]. The hydrothermal method was employed for large-scale production of nitrogen-doped graphene [25,26]. The preparation of nitrogen-doped graphene using hydrothermal methods was realized using different dopants such as melamine [27,28], hydrazine and ammonia [29], ammonium carbonate [30], 1,4-butanediamine [31], ethylenediamine [32], etc. As an example, Kamali and Golsheikh [33] synthesized well-dispersed, nitrogen-doped graphene, through the hydrothermal reduction of graphene oxide in the presence of ammonia. Nitrogen-doped graphene hydrogels were prepared using graphene oxide as raw material and nine amino acids with different acidities as doping agents [34]. Xing et al. [35] have used a simple one-step hydrothermal method by employing hexamethylenetetramine as single carbon and nitrogen source. Depending on the synthesis method, the nitrogen doping level can reach 9–11 at.% [36]. Besides this, the performance of the material is strongly influenced by the number of layers present within the graphene flakes. It has been demonstrated that the electronic structure of graphene changes rapidly with the number of layers, reaching the 3D limit of graphite at 10 layers [37]. Nitrogen-doped graphenes have been intensively investigated for their electrochemical behavior due to their capacity to improve electrical conductivity [36]. N-graphene exhibited enhanced electro-catalytic activity and showed potential applications in electrochemical sensors (8-hydroxy-2′-deoxyguanosine, adenine, guanine, thymine and cytosine) [38,39] and energy devices [40,41].

In this paper, we present for the first time the influence of the reaction time on the morphology, structure and electrochemical properties of nitrogen-doped graphenes. We show that the hydrothermal reaction may produce high quality nitrogen -doped graphene with very low charge-transfer resistance (R_ct_) and large apparent heterogeneous electron transfer rate constant (K_app_). In addition, we originally proved the relationship between these two parameters and the electro-catalytic effect of N-doped graphene towards the detection of cancer biomarker, 8-hydroxy-2’-deoxyguanosine (8-OHdG).

## 2. Materials and Methods

### 2.1. Chemicals and Materials

Potassium ferrocyanide-K_4_[Fe(CN)_6_] and potassium chloride (KCl) were purchased from Merck (Germany). *N*,*N*-dimethylformamide (DMF) was bought from Sigma-Aldrich (Germany). 8-hydroxy-2’-deoxyguanosine (8-OHdG) was purchased from the Cayman Chemical Company. For the synthesis of nitrogen-doped graphene the following chemicals were used: graphene oxide, prepared using a modified Hummers method as previously reported [42], and urea (Alfa-Aesar, Germany).

### 2.2. Instruments

The morphological and structural characterization of the samples was performed with: scanning electron microscope (SU-8230 STEM system, Hitachi, Japan); X-ray powder diffraction (Bruker D8 Advance Diffractometer)(Germany); Fourier transform infrared spectroscopy (Bruker Tensor II spectrometer) (Germany); Raman spectroscopy (JASCO (NRS 3300) spectrophotometer) (USA); and X-ray photoelectron spectroscopy (SPECS spectrometer, equipped with a dual-anode X-ray source Al/Mg, a PHOIBOS 150 2DCCD hemispherical energy analyzer and a multi-channeltron detector) (Germany). Elemental analysis of the synthesized samples was performed with an elemental analyzer—FLASH EA 1112 (Italy).

For electrochemical measurements (cyclic voltammetry—CV, linear sweep voltammetry—LSV, and electrochemical impedance spectroscopy—EIS) a potentiostat/galvanostat instrument (PGSTAT-302N, Metrohm-Autolab B.V., Utrecht, The Netherlands) connected to a three-electrode cell and controlled by PC was employed. The counter electrode was a large area platinum foil (2 cm^2^), while the reference was an Ag/AgCl electrode (3 M KCl). The CV and LSV measurements were generally run from −0.2 to + 0.8 V vs. Ag/AgCl, at various scan rates (2, 5, 10, 15, 20, 30, 40 and 50 mV·s^−1^). The EIS spectra were recorded over 0.1–10^6^ Hz range (10 mV amplitude excitation signal) at the peak potential of each electrode, and the experimental data were fitted using Nova 1.11 software (Metrohm-Autolab B.V., Utrecht, The Netherlands).

### 2.3. Synthesis of Nitrogen-Doped Graphene

**Scheme 1:**
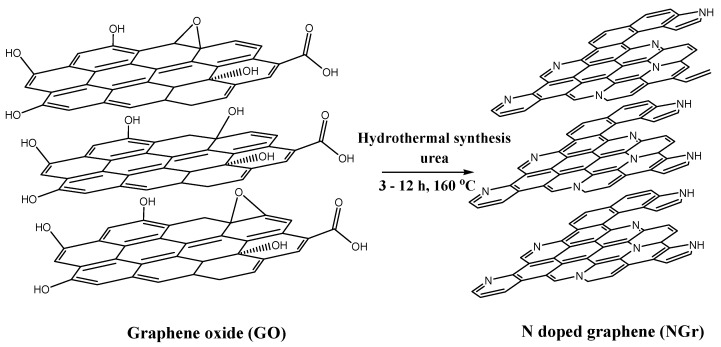
Schematic representation of nitrogen-doped graphene prepared using the hydrothermal method.

Three nitrogen-doped graphene samples were synthesized by the hydrothermal method using urea as doping and reducing agent for graphene oxide (GO), as reported before [43] (Scheme 1). Typically, an appropriate amount of urea was added to 150 mL aqueous dispersion of GO, previously sonicated for 25 min. The mixture was poured into a 250 mL autoclave and placed in the oven at 160 °C for different periods of time (3, 8 and 12 h). The samples were correspondingly denoted NGr-1, NGr-2 and NGr-3. The reduction of GO was observed immediately after the reaction was finished: the well-dispersed brown solution (GO in water) became a clear yellowish solution with a black solid deposited at the bottom of the autoclave. After cooling to room temperature, each sample was filtered, washed with distilled water and dried by lyophilization. Finally, all samples were thermally treated at 300 °C in Ar flow, for 15 min, to remove the excess urea and the residual oxygen-containing groups.

### 2.4. Modification of Glassy-Carbon Electrodes with Graphene-Based Materials

The nitrogen-doped graphenes (NGr-1, NGr-2, NGr-3) and GO were individually dispersed in *N*,*N*-dimethylformamide (1 mg/mL) for three minutes with an ultrasonic finger device (SONICS Vibra-Cell). The obtained graphene suspensions were then used to modify four glassy carbon (GC) electrodes. Before modification, the electrodes were polished on a felt cloth then washed with a large quantity of double-distilled water (20 mL) and dried at room temperature. Next, a volume of 10 μL of graphene suspension (1 mg/mL) was drop-casted onto each GC surface and used for electrochemical investigation, after 24 h drying at room temperature. The modified electrodes were correspondingly denoted as GC/NGr-1, GC/NGr-2, GC/NGr-3, and GC/GO. Before CV or EIS measurements, the bare GC and graphene-modified electrodes were cycled in potassium ferrocyanide solution (10 cycles between −0.2 to +0.65 V vs. Ag/AgCl, with 50 mV/s).

## 3. Results and Discussions

### 3.1. Morphological and Structural Characterization of the Nitrogen-Doped Graphene Samples

**Figure 1:**
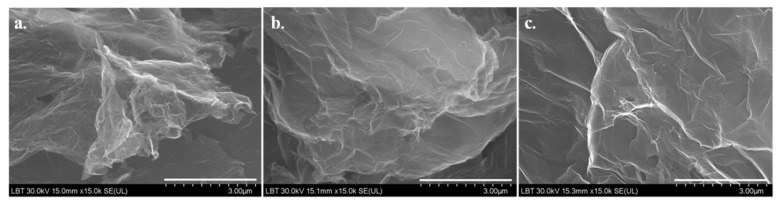
Representative (scanning electron microscopy) SEM images of: NGr-1 (**a**), NGr-2 (**b**) and NGr-3 (**c**); in all images, the scale bar is 3 μm.

The scanning electron microscopy (SEM) technique was employed for the morphological characterization of the nitrogen-doped graphene. Representative SEM images are presented in Figure 1, where one can see that all the samples have highly wrinkled surfaces. Such disordered morphologies imply weak π–π stacking interaction, originating from the doping with nitrogen atoms. The samples are formed by large flakes with a linear size in the range of micrometers.

The elemental analysis of the nitrogen-doped samples gave further indication about the doping level. For NGr-1 and NGr-2 samples where the synthesis time was 3 and 8 h respectively, the heteroatom concentration was around 6.36 wt% (see Table 1). In the case of the NGr-3 sample (12 h reaction time)a higher concentration was obtained, of 6.85 wt%.

Next, the characterization of the nitrogen-doped graphene samples (NGr-1, NGr-2 and NGr-3) was performed using various techniques such as X-ray powder diffraction (XRD), Raman spectroscopy, Fourier-transform infrared spectroscopy (FTIR) and X-ray photoelectron spectroscopy (XPS). Each of them gave valuable information regarding the size of the graphene crystallites, the number of layers and the functionalities attached to the graphene surface.

**Figure 2:**
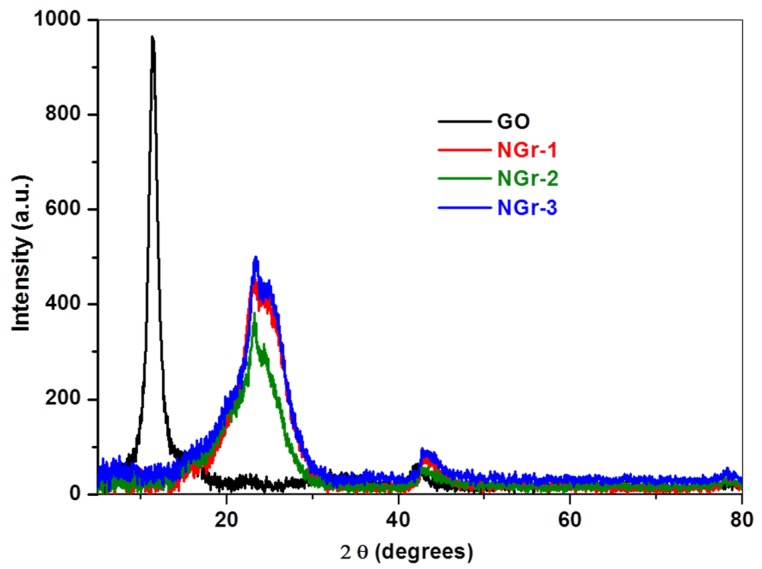
The XRD (X-ray powder diffraction) patterns of GO, NGr-1, NGr-2 and NGr-3 samples.

The X-ray powder diffraction pattern of GO and of the subsequently doped materials NGr-1, NGr-2 and NGr-3 are displayed in Figure 2. One can see that the sharp diffraction peak present at 2θ ~11° and indexed to (001) crystal plane of graphene oxide completely disappears after nitrogen doping and reduction reaction, and a new peak at ~22°, attributed to (002) crystal plane of reduced graphene oxide, is visible. The new peak is broader and has lower intensity due to the disorder induced by the nitrogen atoms into the graphene lattice.

**Figure 3:**
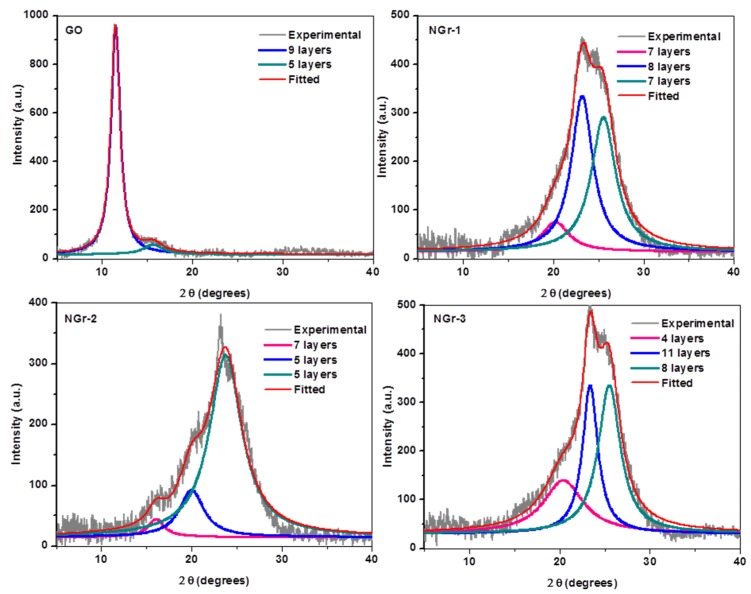
The XRD patterns of GO, NGr-1, NGr-2 and NGr-3 samples and the corresponding deconvoluted peaks.

The XRD experimental data allowed the calculation of the number of layers (*n*) present within graphene oxide and nitrogen-doped graphenes, the interlayer distance (*d*), and the mean crystallite size (*D*) of graphene. For all nitrogen-doped samples, the asymmetric peak from 22° was deconvoluted into three theoretical Gaussian peaks (Figure 3). For each peak, the *D* value was calculated from the full width at half maximum (FWHM) using the Debye-Scherrer equation:

(1) $$D=\frac{0.9*\lambda }{\beta *\cos\theta }$$

where λ is the X-ray wavelength, β is the FWHM expressed in radians, and θ is half the diffraction angle of the peak (in degrees) corresponding to interlayer distance, *d*. The *d* value was found with the Bragg equation (*d = λ/2 sin θ*), while the average number of layers, *n*, was calculated from the relation *n = D/d*. Since the N-doped graphene samples are composed of crystallites having different sizes and number of layers, their contribution (expressed in %) may be determined by dividing the area of each deconvoluted peak to the total area of the diffractogram [44,45] (see Table 2).

From Table 2 it can be seen that the interlayer distance of GO and N-doped graphenes is larger than that of graphite (0.335 nm). The largest value (0.77 nm) is found in GO sample and is attributed to the oxygen functional groups formed after the strong oxidation treatment of graphite. In this case, the average number of layers is 9 (92%).

**Figure 4:**
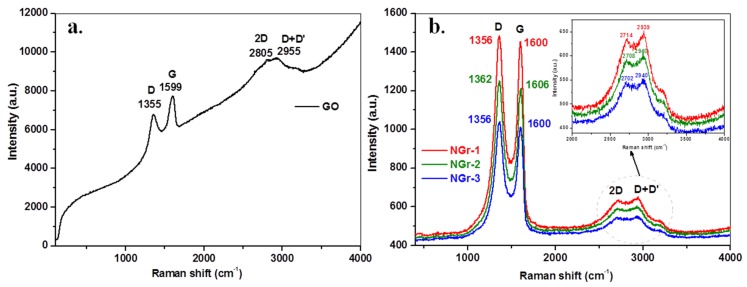
Raman spectra of GO (**a**) and nitrogen-doped graphene samples (**b**): NGr-1 (red); NGr-2 (olive); NGr-3 (blue).

In order to evaluate the degree of structural disorder within graphene oxide and nitrogen-doped graphene samples, Raman spectroscopy was carried out (Figure 4). The high fluorescence signal of GO additionally confirms that the material is highly oxidized, being in good agreement with the XRD pattern. In the nitrogen-doped graphene samples, three important bands are evidenced: the defect (D) band at ~1360 cm^−1^, the graphite (G) band located at ~1598 cm^−1^, and the 2D band at ~2710 cm^−1^ (Table 3).

In addition, the D+D’ band is also present at around 2940–2944 cm^−1^ and appears in graphene-like materials with structural defects, which explain its presence in the nitrogen doped samples [46]. The I_D_/I_G_ ratio is related to the in-plane crystallite size (L_a_), as shown by Equation (2), giving an indication of the defect-free domains [47]:

(2) $$L_{a}\left(\mathrm{nm}\right)=\frac{560}{E_{l}^{4}}{\left(\frac{I_{D}}{I_{G}}\right)}^{-1}$$

where E_l_ represents the laser excitation energy (2.41 eV). The largest *L_a_* value was obtained for GO (17.11 nm) followed by NGr-1 (16.24 nm) and NGr-2 (16.16 nm) samples, which according to elemental analysis had similar amounts of nitrogen (6.36 wt%). For the NGr-3 sample, the L_a_ value is slightly lower (16.12 nm) being in good agreement with the higher doping level (6.85 wt%).

**Figure 5:**
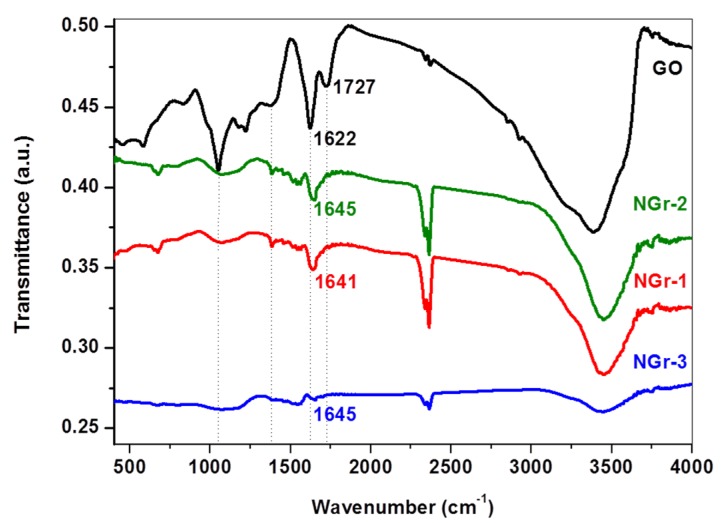
FTIR spectra of GO (black) and of the nitrogen-doped samples NGr-1 (red), NGr-2 (olive) and NGr-3 (blue).

Additionally, FTIR spectroscopy was used to investigate the bonding changes within the carbon atoms after graphene oxide was thermally reduced and doped with nitrogen. The corresponding spectra of GO and of the nitrogen-doped samples NGr-1, NGr-2 and NGr-3 are presented in Figure 5.

In the FTIR spectrum of GO, due to the extensive oxidation, a strong and broad O-H stretching vibration band appears at 3410 cm^−1^ along with the characteristic peak of the C=O stretching vibration at 1727 cm^−1^, the O-H deformation at 1387 cm^−1^, the C-O (epoxy) stretching vibration at 1225 cm^−1^, the C-O (alcoxy) stretching vibration at 1050 cm^−1^ and the epoxy or peroxide group peak at 976 cm^−1^. After hydrothermal reaction, the intensity of the 3391 cm^−1^ peak significantly decreases, while the peak at 1727 cm^−1^, attributed to the C=O groups, disappears almost completely. An important feature is the shifting of the peak from 1622 cm^−1^, correlated to the in-plane vibration of C=C, to 1641-1645 cm^−1^ in the nitrogen-doped samples. This is due to the overlapping of the C=C and C=N vibrations, which gives further evidence of the insertion of nitrogen containing groups [48].

**Figure 6:**
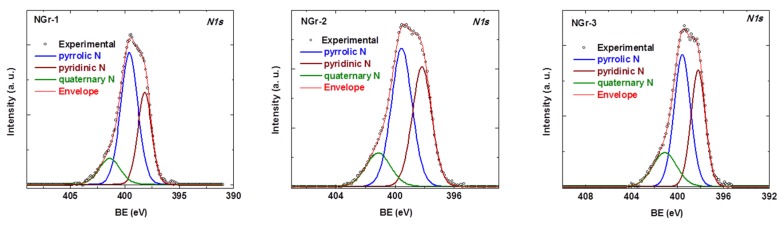
*N 1s* core-level spectra of NGr-1, NGr-2 and NGr-3 samples.

The amount of nitrogen and the nature of the functional groups present at the surface of the obtained materials were determined using XPS. Figure 6 shows the N1s core-level spectra obtained for the prepared samples.

Each spectrum was deconvoluted into three components, approximately at 398.2 eV, 399.5 eV and 401.2 eV, which were attributed to pyridinic-N, pyrrolic-N and graphitic-N [49], respectively. Based on the spectrum deconvolution, it results that the NGr-1 sample predominantly contains pyrrolic-N (55.4 at.%). This percentage decreases with the reaction time, therefore the NGr-2 and NGr-3 samples have smaller amounts of 47.6 and 46.3 at.%, respectively. The NGr-2 sample contains the largest percentage of pyridinic-N (38.6 at.%) as compared to the other investigated samples. The amount of graphitic-N (quaternary) is comparatively lower in all three samples in relation to the other components identified after the spectra deconvolution. This demonstrates that the hydrothermal treatment of GO with urea results in preferential surface functionalization, depending on the reaction time (Table 4), with significant influence on the electrochemical characteristics of N-graphene modified electrodes. The total amounts of nitrogen with respect to that of carbon were determined to be 0.08 (NGr-1), 0.075 (NGr-2) and 0.074 (NGr-3) (estimated by calculating the ratio between C 1s and N 1s peak areas and by considering the sensitivity factors, i.e., nitrogen atoms were supposed to be doped uniformly in the graphitic layers). As can be seen in Table 4, by increasing the reaction time the percentage of pyrrolic-N decreased, while pyridinic-N and graphite-N unevenly increased. Therefore, we suggest that at longer reaction time, some pyrrolic-N is converted to pyridinic-N or graphitic-N. This is due to the fact that during the hydrothermal reaction, urea releases carbimide and NH_3_. Carbimide reacts with hydroxyl groups of GO to form intermediates after dehydration and rearrangement, resulting in more pyrrolic-N. The possible reaction mechanism was published by Shang et al. [50].

Measurements of the high-resolution XPS spectra of *C1s* and *O1s* were also achieved and the results are shown in Table 5.

A comparison of synthetic details of previously reported similar nitrogen-doped graphene is summarized in Table 6.

It is clear that the nitrogen content at the graphene surface within our samples is close to that reported by other groups (Table 6) or even better taking into account that the GO: urea ratio was significantly lower.

### 3.2. Electrochemical Studies

**Figure 7:**
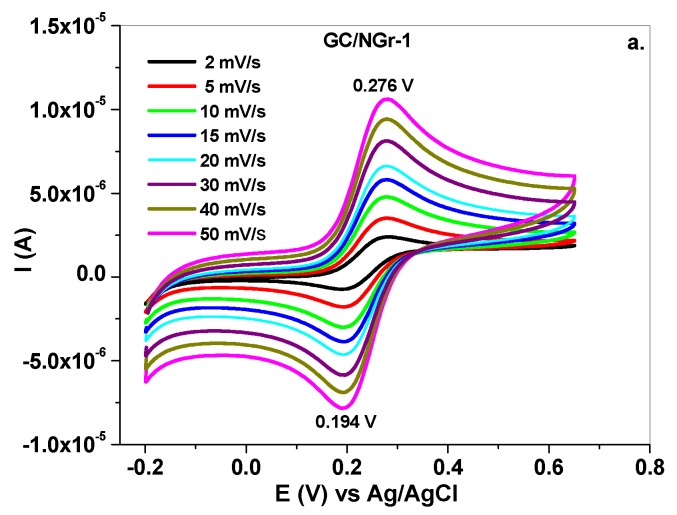
Cyclic voltammograms recorded with different electrodes: GC/NGr-1 (**a**), GC (**b**) and GC/GO (**c**) in 10^−3^ M K_4_[Fe(CN)_6_] (0.2 M KCl supporting electrolyte); scanning rate from 2 to 50 mV/s.

Electrochemical studies regarding the reversibility of the redox process, the charge-transfer resistance and the apparent heterogeneous electron transfer rate constant of bare and graphene-modified electrodes are next presented and discussed. The first experiments were devoted to the calculation of the active area of each electrode. In order to achieve that, CVs were recorded with different scanning rates (from 2 to 50 mV/s) in the presence of a redox indicator, K_4_[Fe(CN)_6_] (10^−3^ M in 0.2 M KCl supporting electrolyte; Figure 7a–c). In the case of all NGr-modified electrodes (see for example GC/NGr-1 in Figure 7a and Table 7) well-defined anodic and cathodic peaks were obtained with a small peak potential separation (ΔE_p_ = 76 mV). In contrast, for the bare GC electrode the two waves were very broad and the peak potential separation large (400 mV) (Figure 7b). GO modified electrode was a special case with a poorly defined electrochemical signal, confirming the GO insulating properties (Figure 7c).

The active area of the bare and NGr-modified electrodes was calculated using the slope of the corresponding (I_peak_) vs. ʋ^1/2^ plot [58], and the values can be found in Table 7, along with other parameters derived from cyclic voltammetry (at a scan rate of 10 mV/s). The smallest area was that corresponding to the GC/GO electrode (0.008 cm^2^), followed by bare GC (0.02 cm^2^). All NGr-modified electrodes had larger surface areas, varying from 0.036 to 0.05 cm^2^. The best characteristics were those of the GC/NGr-1 electrode (A = 0.05 cm^2^; I_pa_/I_pc_~1 and ΔE_p_~76 mV/n), probably due to the highest concentration of pyrrolic-N among all N-doped graphene. On the other hand, for GC and GC/GO electrodes, the redox process can be considered as quasi-reversible (I_pa_/I_pc_ > 1 and ΔE_p_ > 200 mV/n).

Another important parameter calculated from the recorded CVs was the surface coverage with the redox species during the anodic oxidation (Γ*_a_*) [59]:

Γ*_a_ = Q_a_/nFA* (3)

where *Q_a_* is the charge under the anodic peak (Coulomb), *n* is number of transferred electrons (n = 1), *F* is the Faraday number (96485 C/mol) and *A* is the active area (cm^2^).

One can see that the largest surface coverage was obtained for bare GC electrode, followed by N-doped graphene modified electrodes. This is an interesting result which may be explained by the well-known hydrophobicity of nitrogen-doped graphene. In fact, for GC/NGr-1 and GC/NGr-2 electrodes the Γ*_a_* values were similar and slightly lower than that corresponding to GC/NGr-3 (NGr-3 sample had the highest doping level, of 6.85 wt%).

**Figure 8:**
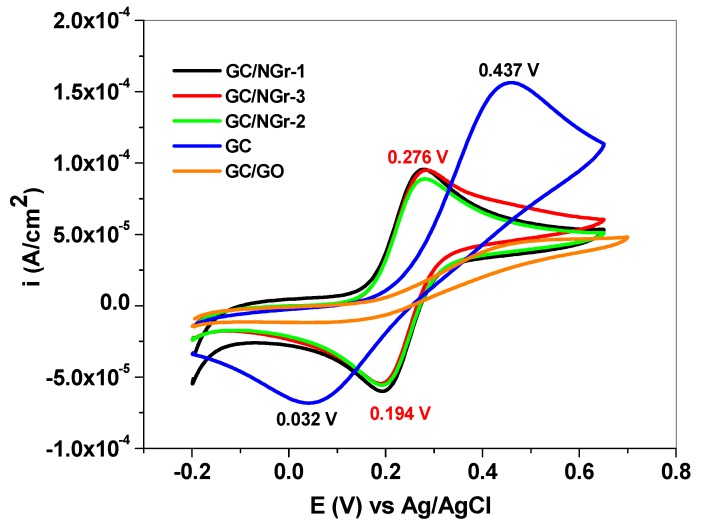
Comparison between the current densities of GC, GC/NGr and GC/GO electrodes in solution containing 10^−3^ M K_4_[Fe(CN)_6_]; 0.2 M KCl supporting electrolyte; 10 mV/s scan rate.

As expected, GC/GO had the lowest surface coverage of 44 × 10^−12^ mol/cm^2^. One of the reasons may be due to various oxygen-containing groups attached to the surface which hinder both the adsorption of redox species and the transfer of electrons. The surface coverage is well correlated with the current density (A/cm^2^) recorded with each electrode (Figure 8). The largest current density was obtained with the bare GC electrode, followed by the NGr-modified electrodes and GC/GO electrode.

Next, the interfacial properties of bare and N-doped graphene modified electrodes were evaluated using electrochemical impedance spectroscopy. The spectra were recorded in 10^−3^ M K_4_[Fe(CN)_6_] (0.2 M KCl supporting electrolyte) at a bias corresponding to the peak potential of each electrode (+0.437 V for GC and GC/GO; +0.276 V for all NGr-modified electrodes) within 0.1–10^6^ Hz frequency range.

**Figure 9:**
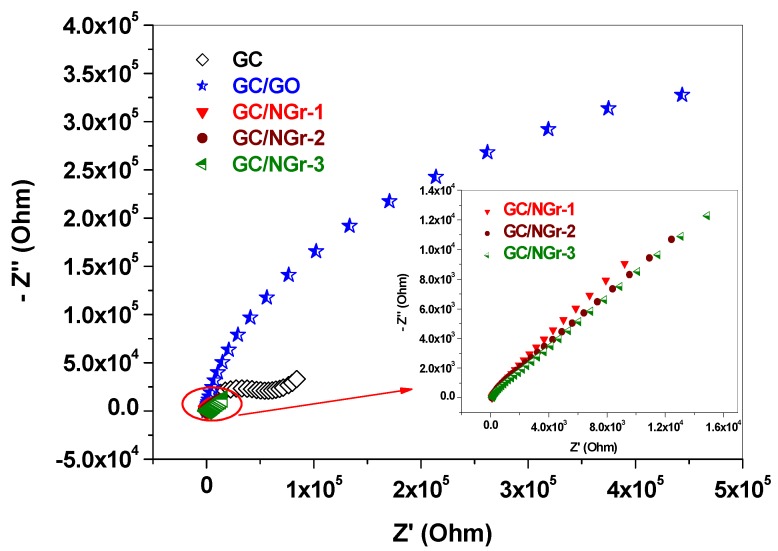
Nyquist plots obtained for GC, GC/GO and NGr-modified electrodes in 10^−3^ M K_4_[Fe(CN)_6_] (0.2 M KCl supporting electrolyte); applied potential: +0.437 V for GC and GC/GO; +0.276 V for GC/NGr; 0.1–10^6^ Hz frequency range.

**Figure 10:**
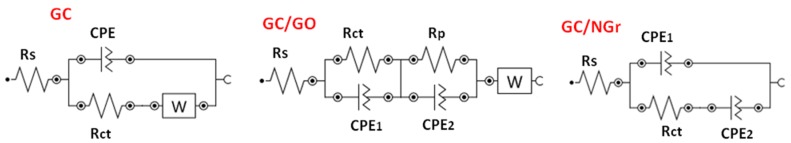
Modified Randles electrical equivalent circuits employed for fitting the experimental EIS data for GC, GC/GO and GC/NGr electrodes.

In Figure 9 are presented the Nyquist plots obtained with GC, GC/NGr and GC/GO electrodes. The bare GC electrode exhibits a semicircle in the high-medium frequencies, characteristic to the charge-transfer resistance (R_ct_), and a straight line at low frequencies, characteristic to the diffusion of the electro-active species towards the electrode/solution interface (Warburg impedance). In the case of the GC/GO electrode, the semicircle is highly extended due to the insulating properties of graphene oxide. In contrast, for the electrodes modified with nitrogen-doped graphene the impedance values decreased by one order of magnitude, both the semicircle and the diffusive line (see the inset in Figure 9). The experimental EIS data were fitted with modified Randles electrical equivalent circuits, presented in Figure 10.

For the bare GC electrode, the best fit was obtained with a circuit containing the solution resistance (R_s_), the charge-transfer resistance at the electrode/solution interface (R_ct_), the Warburg impedance due to the diffusion of ions in solution (Z_W_) and a constant phase element (CPE) that models a non-ideal capacitor.

The *CPE* is expressed by:

(4) $$CPE=\frac{1}{A{\left(j\omega \right)}^{n}}$$

where ω is the frequency, j = √−1, while *n* and *A* are constant parameters that result after fitting the experimental data with the proposed circuit. For n = 0, *CPE* behaves as a resistor, and for n = 1, CPE behaves as a capacitor.

The Warburg impedance appears in the Nyquist plot as a 45^°^ straight line and is expressed by:

(5) $$Zw=\frac{\sigma }{\surd \omega }-\frac{j\sigma }{\surd \omega }$$

where σ depends on the concentrations of the ionic species, the electrode area and the reaction kinetics.

For the electrode modified with graphene oxide (GC/GO), the surface is significantly different from that of bare GC. The successive GO layers deposited on top of the substrate were forming a porous structure where the insertion of ions from solution was hindered by the surface charges. Therefore, the equivalent circuit was a more complex one and contained the solution resistance, two RC parallel circuits (where the capacitances were replaced by CPEs) and the Warburg impedance (Figure 10). One of the resistances was attributed to R_ct_, while the other resistance, R_p_, was attributed to the resistance of the porous structure to ions insertion.

In the case of electrodes modified with nitrogen-doped graphene, the surface is less hydrophilic but still has increased roughness compared with bare GC, and therefore, the best fit was obtained when the Warburg impedance was replaced with CPE, while the other circuit elements were preserved (R_s_, R_ct_ and CPE_1_; Figure 10).

The differences between the three electrical equivalent circuits may be attributed to the characteristic of each electrode/solution interface. Hence, graphene oxide has not only insulating properties but also oxygen-containing groups that repel the negatively charged redox species [Fe(CN)_6_]^3-/4-^] and hinder the transfer of electrons. Consequently, the GC/GO electrode had the largest R_ct_ value of all electrodes (145 × 10^3^ Ohm, see Table 8).

A different situation can be found in the case of electrodes modified with nitrogen-doped graphene. The chemical synthesis led to the formation of few-layer graphene containing 6.36–6.85 wt% of nitrogen, with a different proportion of pyrrolic-N,pyridinic-N and graphitic-N (see the XPS results). According to the literature [40,60], the incorporated nitrogen atoms induced changes at the Fermi level by opening the band gap of graphene and enhancing the charge transfer. Moreover, the carbon atoms which are in close vicinity to the heteroatom may be positively charged due to the higher electronic affinity of nitrogen. As a consequence, the negatively charged species from the solution, [Fe(CN)_6_]^3−^, may easily transfer the electrons. The determined R_ct_ values are indeed significantly lower for all GC/NGr electrodes, in comparison with the bare GC or GC/GO electrode. This is also in excellent agreement with the previous electrochemical measurements, where the CVs recorded with the GC/NGr electrodes exhibits the characteristics of a reversible redox process (see Figure 8).

The value of apparent heterogeneous electron transfer rate constant (K_app_) calculated for each electrode (Equation (6)) [61] gives also valuable information about the charge-transfer at electrode/solution interface (see Table 8).

(6) $$K_{\mathrm{app}}=\frac{\mathrm{RT}}{n^{2}F^{2}{\mathrm{AR}}_{\mathrm{ct}}C}$$

where R is the ideal gas constant (8.314 Joule/(mol·K)); T is the temperature (298 K); F is the Faraday constant (96485 C/mol); *n* is the number of electrons transferred during the redox reaction (*n* = 1); A is the active area of the electrode (cm^2^); R_ct_ is the charge-transfer resistance obtained from the fitted Nyquist plots (Ω); C is the concentration of the redox species (mol/cm^3^).

Its value has the same order of magnitude for GC/GO and bare GC (2.1−2.6 × 10^−4^ cm/s) but is considerably higher for all NGr-modified electrodes (6.55−13.9 × 10^−2^ cm/s). The largest value was that corresponding to NGr-1 material, which had the lowest doping level and the highest concentration of pyrrolic-N, among all nitrogen-doped graphene.

### 3.3. Electrochemical Detection of 8-OHdG with Bare and Graphene-Modified Electrodes

In order to further investigate the electrocatalytic properties of nitrogen-doped graphene and graphene oxide samples, the modified electrodes were tested towards the detection of a cancer biomarker, 8-OHdG. For the beginning, cyclic voltammograms were recorded with each electrode in pH6 PBS solution (scan rate 10 mV/s) and the results are shown in Figure S1 (Supplementary Material). Important information can be obtained from these CVs related to the capacitive current (I_c_), which reflect the charge-storage capability of the synthesized materials. Hence, the graphene samples with the lowest doping level (GC/NGr-1 and GC/NGr-2) have the largest capacitive current, of 1.15 × 10^−7^ A and 1.01 × 10^−7^ A, respectively. In comparison, all the other samples have lower values, such as 5.58 × 10^−8^ A (GC/NGr-3), 1.48 × 10^−8^ A (GC/GO) and 2.39 × 10^−8^ A (GC; see also Table S1). The rest potential (or open-circuit potential) of each electrode was also determined before recording the corresponding CV, and the values are listed in Table S1. The GC/NGr-1 and GC/NGr-2 electrodes have identical rest potential (0.36 V) in good agreement with the doping level. A slightly higher value was obtained for the GC/NGr-3 electrode (0.38 V), while for GC/GO and bare GC the values were lower (0.33 and 0.27 V, respectively).

Next, linear sweep voltammograms were recorded with each electrode in solutions containing various concentrations of 8-OHdG (10^−7^−10^−3^ M) in pH6 PBS supporting electrolyte (scan rate 10 mV/s; see Figure S2a for electrode GC/NGr-1). The corresponding calibration plots are represented in Figure S2b and c. One can observe that there is excellent agreement between the peak current of each electrode and the corresponding R_ct_ and K_app_ values. Hence, among all nitrogen-doped materials, the sample with the lowest R_ct_ and highest K_app_ has the best electrochemical response (GC/NGr-1). It is followed by GC/NGr-3, GC/NGr-2 and bare GC. The poorest electrochemical signal was obtained with the GC/GO electrode, due to the insulating properties of graphene oxide. The electrochemical parameters obtained from the calibration plots (limit of detection, sensitivity and linear range) for bare and graphene-modified electrodes are listed in Table S1, further indicating that the doping level may critically influence the performances of the nitrogen-doped graphene.

## 4. Conclusions

Nitrogen-doped graphenes with various concentrations of heteroatom (6.36, 6.37 and 6.85 wt.%) were prepared using the hydrothermal method with urea as reducing/doping source for graphene oxide. The structural characterization of the synthesized materials revealed that after doping, significant changes occurred in the XRD, Raman and XPS spectra, confirming the presence of heteroatom. For exemplification, the largest value for the in-plane crystallite size *(L_a_)* was obtained for GO (17.11 nm), followed by the NGr-1 (16.24 nm) and NGr-2 (16.16 nm) samples, which according to elemental analysis had similar amounts of nitrogen (6.36 wt%). For NGr-3 sample, the L_a_ value is slightly lower (16.12 nm), being in good agreement with the higher doping level (6.85 wt%). In addition, the EIS studies conducted with NGr-modified electrodes indicated that the sample with the lowest doping level and the highest concentration of pyrrolic-N among all nitrogen-doped graphene (NGr-1) exhibits the best electrochemical parameters: a very small R_ct_ (38.3 Ω), a large K_app_ (13.9 × 10^−2^ cm/s) and a low detection limit (9 × 10^−8^ M) for 8-OHdG.

## Figures and Tables

**Table 1:** Elemental analysis of (graphene oxide) GO and nitrogen-doped graphene samples.

Sample	Reaction Time (Hours)	wt.%			
		C	N	H	O
**GO**	2	52.84	-	1.53	5.63
**NGr-1**	3	80.57	6.36	1.39	11.68
**NGr-2**	8	80.27	6.37	1.09	12.27
**NGr-3**	12	80.45	6.85	1.34	11.36

**Table 2:** Structural parameters obtained from the corresponding XRD pattern of each sample: graphene crystallite size (*D*), interlayer distance *(d*), average number of layers (*n*) and their proportion (*%*) in GO, NGr-1, NGr-2 and NGr-3 samples.

Sample	*2θ* (deg)	*D* (nm)	*d* (nm)	*n*	*%*
**GO**	11.56	6.78	0.77	9	92
	15.65	2.85	0.56	5	8
**NGr-1**	20.23	3.09	0.44	7	11
	23.16	2.88	0.38	8	45
	25.52	2.53	0.35	7	44
**NGr-2**	16.05	4.02	0.55	7	4
	19.99	2.35	0.44	5	16
	23.76	1.82	0.37	5	80
**NGr-3**	20.39	1.66	0.43	4	28
	23.35	4.07	0.38	11	29
	25.47	2.89	0.35	8	43

**Table 3:** Structural parameters obtained from the Raman bands of GO, NGr-1, NGr-2 and NGr-3 samples.

Sample	G (cm^−1^)	I_G_ (a.u.)	D (cm^−1^)	I_D_ (a.u.)	2D (cm^−1^)	I_2D_ (a.u.)	D+D’ (cm^−1^)	I_D+D’_ (a.u.)	I_D_/I_G_	I_2D_/I_G_	L_a_ (nm)
**GO**	1599	1241	1355	1203	2805	600	2955	629	0.97	0.48	17.11
**NGr-1**	1600	1449	1356	1482	2714	635	2939	643	1.022	0.44	16.24
**NGr-2**	1606	1212	1362	1245	2708	594	2940	607	1.027	0.49	16.16
**NGr-3**	1600	1007	1356	1040	2702	548	2940	552	1.03	0.54	16.12

**Table 4:** Elemental composition and relative atomic ratios of GO and identified N species as determined from XPS survey and high resolution *N1s* spectra of the investigated samples.

Sample	Reaction Time (h)	XPS Survey (at.%)			N 1s Core-Level (%)		
		C	O	N	Pyridinic	Pyrolic	Graphitic
**GO**	2	70.7	29.3	-	-	-	-
**NGr-1**	3	85.6	6.8	7.5	31.1	55.4	13.5
**NGr-2**	8	87.2	6.1	6.6	38.6	47.6	13.8
**NGr-3**	12	85.9	7.6	6.4	36	46.3	17.7

**Table 5:** Assignment of each deconvoluted peak based on their binding energies (BE) and atomic concentrations [AC, %] for GO, NGr-1, NGr-2 and NGr-3 samples.

Elements	Binding Energy (eV)/[at.%]				Assignments
	GO	NGr-1	NGr-2	NGr-3	
**C1s**	284.43 (31.2%)	284.5eV (39.6%)	284.4eV (44.4%)	284.4eV (41.8%)	C=C
	285.4 (11%)	285eV (38.9%)	285eV (25.6%)	285.2eV (31.4%)	C-C
	-	286.6eV (13.9%)	286.2eV (19.8%)	286.7eV (17.6%)	C-N
	287.75 (9%)	288.6eV (4.7%)	288.8eV (7%)	289.9eV (6%)	C=O
	289.3 (1.35%)	290.9eV (2.9%)	291.2eV (3.2%)	291.9eV (3%)	O-C=O
**O 1s**	531.8 (4.87%)	530.6eV (42.3%)	530.4eV (33%)	530.4eV (32.9%)	C=O
	532.72 (22.45%)	532.1eV (31.7%)	531.9eV (37.3%)	531.9eV (43.3%)	-OH
	533.4 (0.89)	533.5% (25.9%)	533.1eV (24.8%)	533.1eV (23.7%)	O-C=O

**Table 6:** Comparison of different nitrogen-doped graphene samples prepared from urea and graphene oxide, using the hydrothermal method.

GO: Urea (Weight)	Temperature (°C)	Reaction Time (h)	Nitrogen Content (at.%)	Ref.
1:3	190	12	-	[51]
1:60	180	2–18	7.6	[52]
1:300	180	12	-	[53]
1:300	180	12	11.36	[54]
1:390	180	12	6.05	[55]
1:30	160	3	6.61	[56]
1:30	160	3	5	[57]
1:10	160	3	7.5	This work
1:10	160	8	6.6	
1:10	160	12	6.4	

**Table 7:** The electrochemical parameters of bare, GO- and NGr-modified electrodes.

Electrode	E^0′^ V	ΔE_p_ V	I_pa_ µA	I_pc_ µA	I_pa_/I_pc_	Q_a_ µC	Q_c_ µC	A cm^2^	Γ_a_ pmol/cm^2^
**GC/NGr-1**	0.238	0.076	3.711	−3.554	1.04	0.755	0.648	0.05	156
**GC/NGr-2**	0.236	0.079	3.073	−3.090	0.99	0.656	0.613	0.043	157
**GC/NGr-3**	0.235	0.081	2.698	−2.714	0.99	0.609	0.562	0.036	175
**GC**	0.254	0.366	1.789	−1.493	1.19	0.394	0.536	0.02	204
**GC/GO**	0.318	0.232	0.131	−0.08	1.54	0.034	0.02	0.008	44

Q_a_ = Anodic Peak area; Q_c_ = Cathodic Peak area; A = active area; E^o^’- formal potential; Γ_a_ – surface coverage (pmol/cm^2^).

**Table 8:** The values of R_ct_ and K_app_ for bare and GC modified electrodes.

Electrode	GC/NGr-1	GC/NGr-2	GC/NGr-3	GC	GC/GO
**R_ct_(Ohm)**	38.3	257	112	50.9 × 10^3^	145 × 10^3^
**K_app_ (cm/s)**	13.9 × 10^−2^	2.39 × 10^−2^	6.55 × 10^−2^	2.6 × 10^−4^	2.1 × 10^−4^

## Supplementary Materials

The following are available online at https://www.mdpi.com/1424-8220/20/7/1815/s1, Figure S1: Cyclic voltammograms recorded with bare and graphene-modified electrodes in pH 6 PBS solution; scan rate 10 mV/s, Figure S2: Linear sweep voltammograms recorded with GC/NGr-1 electrode in solutions containing various concentrations of 8-OHdG, 10^−7^–10^−3^ M; pH6 PBS supporting electrolyte; scan rate 10 mV/s (**a**); the corresponding calibration plots for 8-OHdG obtained with bare GC and nitrogen-doped graphene modified electrodes (**b**); the calibration plot for 8-OHdG obtained with GC/GO electrode (**c**). Table S1: Electrochemical parameters obtained from CVs (Figure S1) and calibration plots (Figure S2) for bare and graphene-modified electrodes.
