# Supplementary material for: Nitrogen-Doped Graphene: The Influence of Doping Level on the Charge-Transfer Resistance and Apparent Heterogeneous Electron Transfer Rate
Source: Sensors (Basel). 2020 Mar 25;20(7):1815. doi: 10.3390/s20071815 (PMC7180784; doi:10.3390/s20071815)
Supplement: Supplementary file 1 [file sensors-20-01815-s001.pdf]

Supplementary File

Nitrogen-Doped Graphene: The Influence of Doping Level on the Charge-Transfer Resistance and Apparent Heterogeneous Electron Transfer Rate

Maria Coros [1,*], CodrutaVarodi [1], Florina Pogacean [1], Emese Gal [2] and Stela M. Pruneanu [1,*]

1 National Institute for Research and Development of Isotopic and Molecular Technologies, Donat Street, No. 67-103, 400293, Cluj-Napoca, Romania; codruta.varodi@itim-cj.ro (C.V.); florina.pogacean@itim-cj.ro (F.P.)

2 Department of Chemistry and Chemical Engineering, Hungarian Line of Study, Babes-Bolyai University, 11 Arany János St., 400028, Cluj-Napoca, Romania; gal.emese.81@gmail.com

* Correspondence: maria.coros@itim-cj.ro (M.C.); stela.pruneanu@itim-cj.ro (S.M.P.)

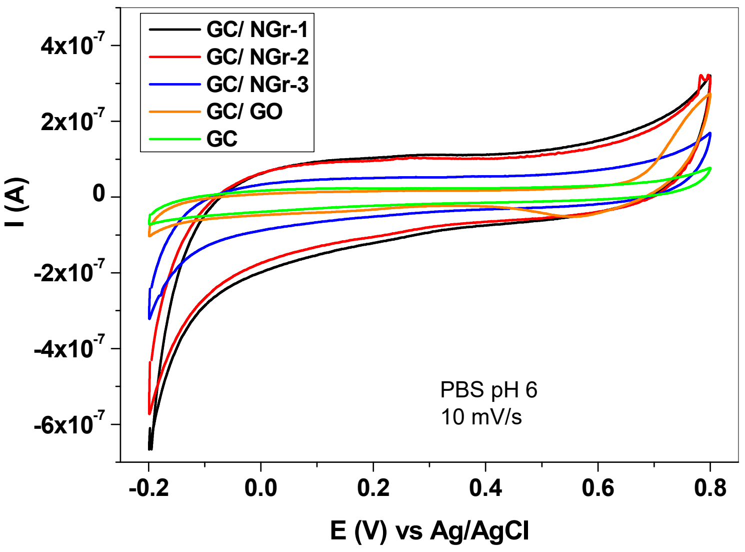

Figure S1. Cyclic voltammograms recorded with bare and graphene-modified electrodes in pH 6 PBS solution; scan rate 10 mV/s.

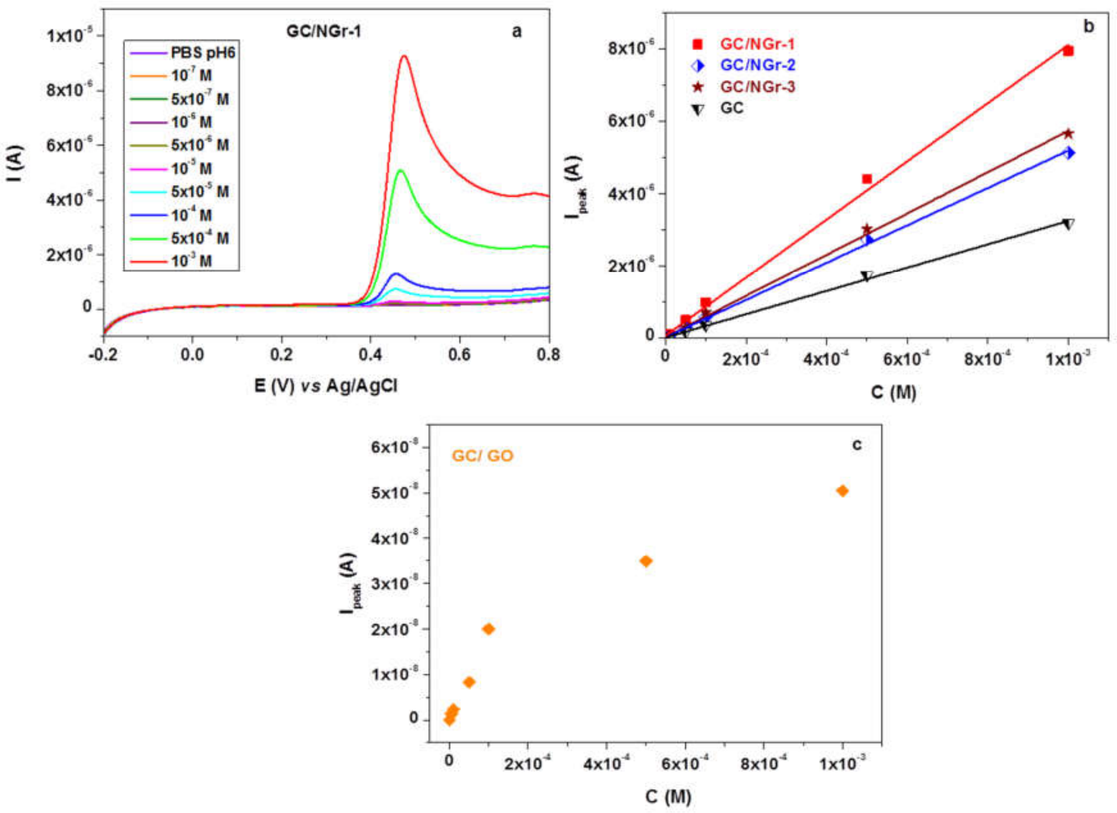

Figure S2. Linear sweep voltammograms recorded with GC/NGr-1 electrode in solutions containing various concentrations of 8-OHdG, 10^{-7} – 10^{-3} M; pH6 PBS supporting electrolyte; scan rate 10 mV/s (**a**); the corresponding calibration plots for 8-OHdG obtained with bare GC and nitrogen-doped graphene modified electrodes (**b**); the calibration plot for 8-OHdG obtained with GC/GO electrode (**c**).

Table S1. Electrochemical parameters obtained from CVs (Figure S1) and calibration plots (Figure S2) for bare and graphene-modified electrodes.

Electrode	Ic (A)	Rest Potential (V)	Sensitivity (mA/M)	LOD (M)	Linear Range (M)
GC/NGr-1	1.15×10^{-7}	0.36	8	9×10^{-8}	3×10^{-7} – 1×10^{-3}
GC/NGr-2	1.01×10^{-7}	0.36	5.1	1.5×10^{-7}	5×10^{-7} – 1×10^{-3}
GC/NGr-3	5.58×10^{-8}	0.38	5.7	1.5×10^{-7}	5×10^{-7} – 1×10^{-3}
GC/GO	1.48×10^{-8}	0.33	0.19	1.5×10^{-6}	5×10^{-6} – 1×10^{-4}
GC	2.39×10^{-8}	0.27	3.2	1.5×10^{-7}	5×10^{-7} – 1×10^{-3}
